# Longitudinal 12-Month Follow-Up of a Male Infant with *CYP21A2* Compound Heterozygous Genotype in China: A Case Report

**DOI:** 10.1055/a-2647-4369

**Published:** 2025-07-18

**Authors:** Yi Yin, Xinyue Huang, Yun Shi, Cheng Huang, Jian Yu, Qingsong Liu

**Affiliations:** 1 Department of Prenatal Diagnosis, Chengdu Women's and Children's Central Hospital, School of Medicine, University of Electronic Science and Technology of China, Chengdu, China; 2 Department of Neonatology, Chengdu Women's and Children's Central Hospital, School of Medicine, University of Electronic Science and Technology of China, Chengdu, China; 3 Department of Medical Laboratory, Chengdu Women's and Children's Central Hospital, School of Medicine, University of Electronic Science and Technology of China, Chengdu, China; 4 Department of Neonatal Disease Screening, Chengdu Women's and Children's Central Hospital, School of Medicine, University of Electronic Science and Technology of China, Chengdu, China

**Keywords:** 21-hydroxylase deficiency, hyponatremia and hyperkalemia, hyperpigmentation, molecular genetic diagnosis, infant, timely therapeutic intervention, follow-up

## Abstract

**Background:**

Congenital adrenal hyperplasia (CAH), predominantly caused by 21-hydroxylase deficiency (21-OHD), arises from mutations in *CYP21A2* . This frequently occurs via gene conversion events between *CYP21A2* and its pseudogene, leading to impaired 21-hydroxylase activity and subsequent CAH manifestations.

**Case Description:**

We encountered a case of classic CAH, characterized by electrolyte imbalances (hyponatremia: 125.10 mmol/L; hyperkalemia: 7.06 mmol/L), hyperpigmentation, and markedly elevated endocrine marker levels (17-hydroxyprogesterone: 319.91 nmol/L; adrenocorticotropic hormone: 611.00 pg/mL) in a male neonate. Through genetic diagnostics, we identified a maternal-derived deletion of *CYP21A2* exons 1–7 combined with paternal-originated compound heterozygous mutations (c.293-13A/C>G in intron 2 and c.332_339 deletion in exon 3). Implementation of early genetic diagnosis revealed 21-OHD, and immediate therapeutic intervention was initiated within 11 days after the birth of the patient. Long-term treatment, including oral hydrocortisone, fludrocortisone, and 0.9% sodium chloride, provided effective clinical control and management, as determined by longitudinal follow-up monitoring of serum electrolyte profiles, endocrine function, and physical development.

**Conclusion:**

This case provided critical insights into the genotype–phenotype correlations of classic 21-OHD. Our findings will contribute to precision medicine for managing this rare endocrine disorder during critical infancy periods, and emphasize the need for comprehensive genetic diagnostics and educational values for neonatal 21-OHD care.

Congenital adrenal hyperplasia (CAH) comprises a group of autosomal recessive disorders, with approximately 90 to 95% 1 of cases attributable to 21-hydroxylase deficiency (21-OHD) caused by *CYP21A2* mutations. 21-OHD is characterized by cortisol deficiency with or without aldosterone deficiency and androgen excess. Based on the severity of 21-OHD, CAH is classified into classic and non-classic forms, with a strong emphasis on genotype–phenotype correlations. 2 The classic form is further subdivided into the salt-wasting (SW) and simple virilizing (SV) forms. In the SV form, the primary clinical manifestations include virilization of the external genitalia in female newborns, as exemplified by a reported case of a 17-year-old girl presenting with virilized external genitalia and primary amenorrhea, 3 and precocious puberty in boys attributable to androgen overproduction. By contrast, untreated SW caused by aldosterone deficiency can lead to life-threatening complications such as hypovolemia, shock, hyponatremia, hyperkalemia, dehydration, and adrenal crisis. 1 4

21-OHD is a genetic disorder characterized by the impaired biosynthesis of cortisol and aldosterone, accompanied by the accumulation of 17-hydroxyprogesterone (17-OHP) and adrenal androgens. 5 Most classic CAH cases are identified during infancy through the detection of 17-OHP levels in newborn screening programs. In China, the implementation of nationwide newborn screening for CAH commenced in the 1990s, and universal coverage has been achieved across almost all counties. A comprehensive meta-analysis of newborn CAH screening data in China revealed an overall incidence rate of 0.43 per 10,000 live births (95% confidence interval = 0.39–0.48). 6 Notably, significant regional variations in disease prevalence were observed, with Zhejiang, Guangdong, Hubei, and Shaanxi provinces demonstrating lower incidence rates than the national average, whereas other regions exhibited higher prevalence rates than the national estimate. 6

Meanwhile, 21-hydroxylase is encoded by *CYP21A2* , located on the short arm of chromosome 6. *CYP21A1P* , a homologous pseudogene, is situated approximately 30 kb upstream of the functional gene, and it shares >95% sequence homology with *CYP21A2* while maintaining 65 nucleotide differences. 7 The close proximity and high sequence similarity between *CYP21A2* and *CYP21A1P* make these genes prone to frequent recombination events, resulting in the transfer of pseudogene-derived mutations to the functional gene. These recurrent mutations include large deletions/gene conversions, as well as specific point mutations, such as p. Pro31Leu, c.293-13A/C>G, p. Gly111ValfsTer21, p. Ile173Asn, E6 cluster mutations, p. Val282Leu, p. Leu308PhefsTer6, p. Gln319Ter, and p. Arg357Trp. 2 8 More than 200 pathogenic or likely pathogenic variants in *CYP21A2* have been identified. 9 In this study, we presented a case with longitudinal follow-up from birth to 12 months of age in which comprehensive genetic analysis through whole-exome sequencing (WES), Sanger sequencing, and multiplex ligation-dependent probe amplification (MLPA) revealed compound heterozygosity in *CYP21A2* . The patient inherited a large deletion spanning exons 1–7 from the maternal allele and compound heterozygous mutations from the paternal allele. Although the patient was diagnosed with 21-OHD, his phenotypes were controlled effectively. In this report, we described the genetic profile associated with the clinical manifestations of hyperpigmentation and elevated adrenocorticotropic hormone (ACTH) levels and 17-OHP concentrations, as well as the long-term clinical outcomes.

## Case Presentation

The index case was a male infant, the first child of non-consanguineous parents, delivered via cesarean section at 39 ^3/7^ weeks of gestation with a birth weight of 3,700 g. The pregnancy was uneventful. At 7 hours after birth, the newborn was admitted to another hospital presenting with “shortness of breath for 18 minutes.” Physical examination revealed no significant abnormalities excluding generalized hyperpigmentation, notably in the scrotum and penis. The infant exhibited normal male external genitalia, and he did not experience any episodes of hypoglycemia during the neonatal period. Initial blood electrolyte analysis indicated elevated serum potassium and reduced sodium levels, but no specific values were documented. His serum cortisol level was 17.33 μg/dL. Based on these clinical findings, a provisional diagnosis of CAH was made. However, the family history was negative for CAH. During hospitalization, the infant developed abdominal distension, which was managed with a 2-day regimen of fasting, omeprazole for acid suppression, enema, and intravenous nutrition. Following this treatment, normal feeding was successfully resumed. Abdominal ultrasonography revealed distension, with evidence of multiple and slightly dilated intestinal loops, and a left colonic diameter of approximately 2.1 cm.

At 11 days old, he was admitted to our hospital for CAH. At the first evaluation in our hospital, the infant exhibited normotensive anterior fontanel and hyperpigmentation of the skin, specifically the armpit, perineum, and scrotum, with normal testis feel in the scrotum. The laboratory examination results are presented in Table 1 . The patient's serum electrolytes results included a high potassium level (7.06 mmol/L [normal range, 4.2–5.9 mmol/L]), low sodium (125.10 mmol/L [135–150 mmol/L]), low calcium (1.09 mmol/L [2.1–2.8 mmol/L]) and chlorine levels (96.60 mmol/L [100–116 mmol/L]). The patient also exhibited extremely high level of ACTH (611.00 pg/mL [0–46 pg/mL]), testosterone (TSTO; 713.37 ng/dL [0–30 ng/dL]), androstenedione (AND; >10.00 ng/mL [0.6–3.1 ng/mL]), and 17-OHP (319.91 nmol/L [0–30 nmol/L]), whereas his dehydroepiandrosterone (DHEAS) level was normal (291.00 μg/dL [80–560 μg/dL]). Notably, the patient's serum 17-OHP level was consistent with the newborn screening value (312.13 nmol/L), further supporting the diagnosis of CAH.

**Table 1:** Laboratory examination results of the case by follow-up for 12 months

Age	Cut-off value	At admission	At discharge		Follow-up				
		11 days	23 days		1 month	2 months	4 months	6 months	9 months	12 months
AND (ng/mL)	0.6–3.1	**>10.00**	**>10.00**	1.90	**<0.30**	**<0.30**	**<0.30**	**<0.30**	**<0.30**
ACTH (pg/mL)	0–46	**611.00**	**121.00**	**175.00**	–	**57.60**	9.58	**376.00**	–
TSTO (ng/dL)	<7.00	**713.37**	**94.24**	–	–	–	–	<7.00	**15.63**
17-OHP (nmol/L)	0–30	**319.91**	**158.51**	8.30	5.48	–	7.10	**133.71**	–
DHEAS (μg/dL)	80–560	291.00	60.90	**19.00**	**<15.00**	**<15.00**	**<15.00**	**<15.00**	**<15.00**
Potassium (mmol/L)	4.2–5.9	**7.06**	4.73	5.47	4.66	**3.40**	**3.48**	5.47	4.84
Sodium (mmol/L)	135–150	**125.10**	136.40	138.20	139.60	140.70	140.00 (134–143)	133.80 (134–143)	134.40 (134–143)
Chlorine (mmol/L)	100–116	**96.60**	104.20	101.20	105.10	100.80	103.90 (98–110)	98.70 (98–110)	99.50 (98–110)
Calcium (mmol/L)	2.1–2.8	**1.09**	2.63	2.59	2.58	2.73	2.54	2.75	2.47
Cortisol (8 a.m.) (μg/dL)	5.27–22.45	–	–	–	–	–	–	**1.59**	**2.82**

Abbreviations: 17-OHP, 17-hydroxyprogesterone; ACTH, adrenocorticotropic hormone; AND, androstenedione; DHEAS, dehydroepiandrosterone sulfate; TSTO, testosterone.

“–” means no data available. The reference range was changed for sodium (134–143 mmol/L) and chlorine (98–110 mmol/L) at the ages of 6, 9, and 12 months.

Note: Bold numerical values indicate values outside the reference range.

Based on the laboratory findings, a comprehensive management plan was implemented upon admission, including the maintenance of body warmth, continuous ECG monitoring, meticulous skin and oral care, and appropriate feeding regimens. For hyperkalemia, intravenous calcium, insulin–glucose solution, and sodium bicarbonate were administered to reduce serum potassium levels and prevent myocardial toxicity. Hormonal therapy with intravenous hydrocortisone (oral hydrocortisone after 3 days) and oral fludrocortisone was initiated 2 days after admission. In addition, hyponatremia was addressed with oral 10% sodium chloride supplementation (15 mL in milk). Color Doppler echocardiography revealed a left-to-right shunt caused by a patent foramen ovale with normal left ventricular function. Cranial ultrasound revealed bilateral subependymal cysts (right: 1.5 cm × 0.5 cm; left: 0.8 cm × 0.5 cm), which resolved by 4 months of age. Abdominal ultrasound confirmed bilateral adrenal gland enlargement with morphological abnormalities, supporting the diagnosis of CAH.

**Fig. 1:**
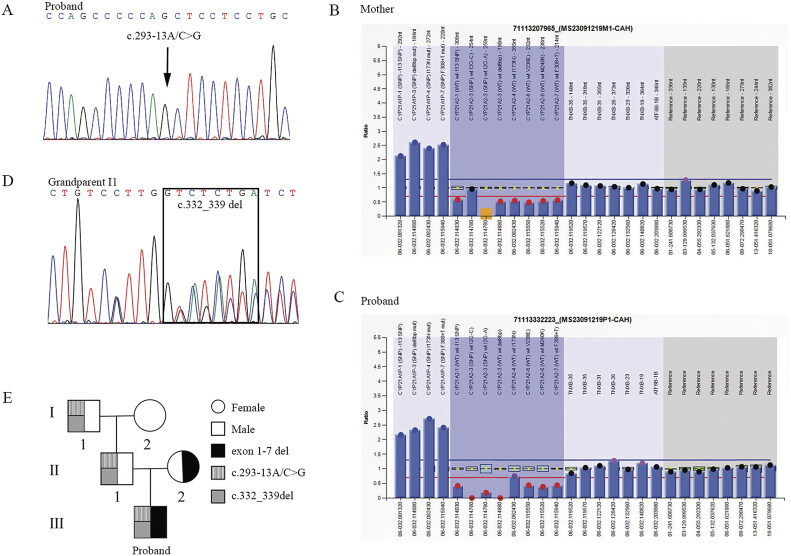
The genetic analysis of *CYP21A2* in the family. ( **A** ) Sanger sequencing validation of the c.293-13A/C>G variant in the proband. ( **B, C** ) MLPA analysis revealed a heterozygous deletion spanning exons 1–7 of *CYP21A* 2 in both the proband ( **C** ) and the mother ( **B** ). ( **D** ) Sanger sequencing confirmation of the c.332_339 deletion in paternal grandparent I1. ( **E** ) Pedigree analysis of *CYP21A2* mutations in the family. Paternal grandparent I1 carried both c.293-13A/C>G and c.332_339 deletion in cis, while I2 was normal. Individual II2 carried the exons 1–7 deletion, and II1 was compound heterozygous for c.293-13A/C>G and c.332_339 deletion (in cis). The proband inherited the exons 1–7 deletion from the mother (II2) and the compound heterozygous c.293-13A/C>G and c.332_339 deletion from the father (II1). MLPA, multiplex ligation-dependent probe amplification.

Furthermore, molecular genetic diagnosis was performed using WES of the peripheral blood leukocytes from this infant. Proband-only WES identified the homozygous typical *CYP21A2* variant c.293-13A/C>G (in intron 2; Table 2 ), which was classified as a pathogenic variant using standard PM3_VeryStrong + PS3 + PP4 according to the American College of Medical Genetics and Genomics (ACMG) guidelines 10 and confirmed by Sanger sequencing ( Fig. 1A ). Parental genotyping revealed that the father was a heterozygous carrier of the c.293-13A/C>G variant, whereas the mother was wild-type ( Supplementary Data ). Notably, Sanger sequencing of the father revealed double peaks from 51-bp downstream of the c.293-13A/C>G locus ( Supplementary Data [available in the online version only]), suggesting an additional variant. Based on the polymorphism of *CYP21A2* , specific MLPA (P050, MRC-Holland, Amsterdam, the Netherlands) for *CYP21A2* was conducted for both the proband and his mother. MLPA identified a large deletion spanning exons 1–7 of *CYP21A2* in the mother, confirming a single-copy loss in this region ( Fig. 1B ). In addition, the proband exhibited a heterozygous deletion of exons 1–7 in *CYP21A2* (pathogenic: PVS1 + PM3_Strong +PM2), consistent with inheritance from the mother ( Fig. 1C and Table 2 ). Additionally, a second pathogenic variant (PVS1 + PM3_VeryStrong + PM2), namely c.332_339 deletion (p. Gly111Valfs*21, in exon 3), was identified in the proband, corresponding to the father's double peaks and indicating a compound heterozygous state attributable to exons 1–7 deletion ( Table 2 ). The results of Sanger sequencing for both the proband and parent are presented in Supplementary Data . The paternal origin of the c.332_339 deletion variant was further validated by genotyping the paternal grandparents ( Fig. 1D ). The result illustrated that the c.332_339 deletion was inherited from grandparent I1, whereas grandparent I2 was wild-type. The family pedigree is summarized in Fig. 1E . Both the c.293-13A/C>G and c.332_339 deletion loci were inherited in cis. The Sanger sequencing results for the two variants in the grandparents are presented in Supplementary Data .

**Table 2:** Genetic analysis results of *CYP21A2* variants identified by whole-exome sequencing and multiplex ligation-dependent probe amplification of the proband

Gene symbol	Location (GRCH38)	Mutation	gnomAD MAF	ACMG variant grade	Disease	Inheritance pattern	Zygote type
*CYP21A2*	Chr6: 32039081	NM_000500.9: exon3: c.293-13A/C>G	0.00226067	Pathogenic: PM3_VeryStrong +PS3 + PP4	Adrenal hyperplasia, congenital, due to 21-hydroxylase deficiency/hyperandrogenism, non-classic type, due to 21-hydroxylase deficiency [MIM: 201910]	AR	Homozygous (parental)
	Chr6: 32039133-32039140	NM_000500.9: exon3: c.332_339 deletion (p. Gly111Valfs*21)	–	Pathogenic: PVS1 + PM3_VeryStrong + PM2			Homozygous (parental)
	Chr6	NM_000500.9: exon (1–7) deletion	–	Pathogenic: PVS1 + PM3_Strong + PP4 + PM2			Heterozygous (maternal)

Abbreviations: ACMG, American College of Medical Genetics and Genomics; LOF, loss-of-function.

PM3_VeryStrong: For recessive disorders, detected in trans with a pathogenic variant, and has been reported in the literature that this variant was detected in several patients with congenital adrenal hyperplasia (CAH) along with other variants, and the homozygous variant has been detected in multiple patients with CAH. PS3: Well-established in vitro or in vivo functional studies supportive of a damaging effect on the gene or gene product. PP4: Patient's phenotype or family history is highly specific for a disease with a single genetic etiology. PM2: 2 Absent from controls (or at extremely low frequency if recessive) in Exome Sequencing Project, 1000 Genomes Project, or Exome Aggregation Consortium. PVS1: Null variant (nonsense, frameshift, canonical ±1 or 2 splice sites, initiation codon, single or multiexon deletion) in a gene where LOF is a known mechanism of disease.

At discharge, the infant's serum electrolyte levels (potassium, sodium, calcium, and chloride) had normalized, and his serum ACTH, TSTO, and 17-OHP levels were significantly lower than those at admission. He was discharged on a regimen of oral hydrocortisone (1 mg three times daily), fludrocortisone (0.05 mg twice daily), and 0.9% sodium chloride (15 mL three times daily), with adjustments based on the results of follow-up evaluations. In addition, monthly follow-up visits for the first 3 months and quarterly thereafter. At 3 months of age, the patient's hyperpigmentation was limited to the scrotal region only. His laboratory findings during the 12-month follow-up are summarized in Table 1 . The patient's serum electrolytes remained stable from 1 to 12 months of age. Although his potassium level slightly fluctuated at 4 and 6 months of age, it remained within the normal range at the next two assessments (at 9 and 12 months old). The indicators of AND and DHEAS levels were consistently below the lower cutoff from 2 to 12 months of age. The patient's ACTH levels remained elevated except at 6 months, whereas his 17-OHP levels were within the normal range at all time points, excluding at 9 months. TSTO and morning cortisol levels (8 a.m.) were measured only at 9 and 12 months. His TSTO level was within normal limits at 9 months, whereas his cortisol remained below the lower detection limit. Then, he continued to follow-up at our Pediatric Health Care Center, demonstrating normal physical development, including head circumference, height, and weight. Ongoing monitoring of his physical and laboratory parameters has been recommended to optimize the management of CAH.

## Discussion

Our case illustrates the classic presentation of CAH, which was characterized by hyponatremia, hyperkalemia, elevated 17-OHP and ACTH levels, and hyperpigmentation, as well as a risk of adrenal crisis. Molecular genetic diagnoses confirmed compound heterozygous mutations in *CYP21A2* , comprising a c.293-13A/C>G variant and a c.332_339 deletion (p. Gly111Valfs*21) variant inherited in cis from the father, along with a large deletion spanning exons 1–7 inherited from the mother. Both parents were heterozygous carriers, eventually resulting in loss of 21-hydroxylase and CAH development in the proband. Molecular genetic techniques, including WES, MLAP, and Sanger sequencing, were employed to diagnose CAH in this male infant with normal male external genitalia, consistent with the genetic findings. According to the genotype − phenotype correlations, all three variants were classified as pathogenic and associated with the severe SW form. 11 12 However, the patient did not develop severe hyponatremia or dehydration because treatments were initiated at an early age. Over the 1-year follow-up period, the patient received daily treatment with hydrocortisone and fludrocortisone, which reduced high androgen levels and replaced adrenal functions. Through this early intervention and treatment, normal plasma volume and physiological balance were maintained to prevent adrenal crisis attributable to adrenal insufficiency. In addition, he underwent continuous monitoring through regular physical examinations, endocrine function assessments, and blood electrolyte measurements, which ensured effective disease management. The long-term dosage of hydrocortisone and fludrocortisone can also be adjusted according to the results of regular check-ups. Therefore, a newborn screening program can detect 17-OHP levels within 72 hours after birth in newborns, which can improve the diagnosis rate and permit immediate treatment for classic CAH.

Although newborn screening for 17-OHP is an effective strategy for the early detection of classic CAH, its utility is limited by high rates of false positives and negatives. These inaccuracies arise from various influencing factors, including gender, birth weight, postnatal age, and methodological differences. 13 In China, 17-OHP detection primarily relies on enzyme-linked immunosorbent assay or dissociation-enhanced lanthanide fluorescence immunoassay, both of which are associated with high false-positive rates and low positive predictive values. 6 By contrast, secondary screening using liquid chromatography-tandem mass spectrometry (LC-MS/MS) has demonstrated significantly improved sensitivity and specificity for CAH diagnosis. In prior research, LC-MS/MS in the secondary screening of raw dried blood spots significantly increased the positive predictive value of CAH screening to 25 − 70%, 14 15 indicating the potential of LC-MS/MS to enhance the accuracy and reliability of CAH screening programs.

As previously described, the high recombination rate (gene conversion) between *CYP21A2* and its pseudogene ( *CYP21A1P* ) contributes to the high frequency of mutations in *CYP21A2* . Variants of c.293-13A/C>G (intron 2: g.655C/A>G) and c.332_339 deletion (exon 3: g.707_714, p.Gly111Valfs*21) are derived from *CYP21A2* and its pseudogene. The c.293-13A/C>G variant, associated with abnormal splicing attributable to the activation of an upstream cryptic splice acceptor site, results in fewer than 5% of residual 21-hydroxylase enzyme activity. 8 Meanwhile, this variant is the most common mutation in China, 16 17 18 as well as in populations from South America, North America, and Europe. 16 In addition, the c.293-13A/C>G variant is the most frequent mutation in cohorts in southern China, specifically compared with different regions in China, including Southeast Asia. 18 19 20 Conversely, the c.332_339 deletion, an 8-bp frameshift deletion in exon 3, leads to a premature stop codon and complete loss of 21-hydroxylase activity, 5 but it occurs less frequently than c.293-13A/C>G. 18 Both variants exhibited strong genotype–phenotype correlations, and they were associated with classic CAH and female genital ambiguity. 2 8 16 21 Additionally, large deletions spanning exons 1–7 of *CYP21A2* have been reported as high frequency mutation in Chinese populations, including a small cohort of 37 patients with CAH 17 and a larger cohort of 166 patients with classical 21-OHD from southern China. 18 These findings underscore the genetic heterogeneity and regional variability of *CYP21A2* mutations in classical CAH.

In this case, we initially utilized WES for genetic diagnosis, identifying a homozygous *CYP21A2* variant (c.293-13A/C>G) in the proband. However, the scope of WES in detecting *CYP21A2* polymorphisms is limited. First, WES only detects the exonic region and ±20-bp intronic region, whereas the variants in introns and large deletions across exons (such as exon 1–7 deletion in our case) cannot be effectively detected. Meanwhile, it is difficult to identify the real mutations existing in *CYP21A2* or *CYP21A1P* , highlighting the risk of missing detections. Considering these limitations, we further employed MLPA and Sanger sequencing to confirm the diagnosis. Through this approach, two additional deletions (c.332_339 deletion and exon 1–7 deletion) were identified in the proband. Sanger sequencing results are accurate, reliable, and highly sensitive. However, it is difficult to detect gene copy number variation, short read length, and small throughput using this technique, and it is not suitable for screening structural variations, such as deletions or duplications. 22 However, long-read sequencing (LRS, also known as single-molecule real-time sequencing) has emerged as a powerful tool for the genetic diagnosis of rare diseases, including CAH and spinal muscular atrophy. LRS offers the unique advantage of generating reads exceeding 200 kb in length without the need for PCR amplification, thereby reducing the rate of mismatches during amplification. 23 24 A distinctive feature of LRS is its capability to determine the allelic configuration (in trans or cis) without a parental comparison, thereby enhancing the diagnostic precision and efficiency. *CYP21A2* comprises 10 exons. However, MLPA is limited to probes targeting exons 1–7, and interference by the pseudogene can affect the accuracy of the probes, which makes it difficult to find mutations across exons 8–10 and decreases the reliability of the results. Moreover, MLPA cannot detect copy number neutral inversions or translocations. By contrast, LRS offers significant advantages in detecting mutant events within exons 8–10, and greater utility in detecting large segmental variations such as structural deletions, duplications, inversions and translocations, and microtransformations. Although LRS has incomparable advantages in detecting genetic structural variations, its high economic cost limits its wide application in clinical practices.

Prenatal diagnosis plays a pivotal role in the early identification and management of CAH, particularly in families with a known history of the disorder. In this case, the identification of compound heterozygous mutations in *CYP21A2* in the proband highlighted the potential utility of prenatal genetic testing in at-risk families, although the parent denied a family history of CAH. Prenatal diagnosis of CAH can be achieved through chorionic villus sampling or amniocentesis, followed by molecular genetic analysis of *CYP21A2* . 25 26 This approach permits the early detection of affected fetuses, thereby enabling timely counseling and intervention. For families with a history of CAH, prenatal diagnosis provides critical information for reproductive decision-making and pregnancy management. In cases where an affected fetus is identified, prenatal treatment with dexamethasone can be considered to mitigate the virilization of external genitalia in female fetuses, a hallmark of classical CAH. 27 Furthermore, prenatal diagnosis facilitates the preparation of health care teams for the immediate postnatal management of affected newborns, including the prompt initiation of glucocorticoid and mineralocorticoid replacement therapy to prevent life-threatening adrenal crises. 9

Early initiation of treatment, including glucocorticoid and mineralocorticoid replacement therapy, prevented the development of severe hyponatremia, dehydration, and adrenal crisis in the patient. Prior research identified four patients as 21-OHD via prenatal genetic diagnosis in four patients born at term. Three patients were boys, none of whom developed SW phenomenon or dehydration because they were treated with hydrocortisone, 9α-fludrocortisone, and sodium chloride starting at a mean of 3.7 days after birth (range, 2–7 days). 9 Meanwhile, the female infant patient exhibited ambiguous genitalia because of increased exposure to adrenal androgens in utero. In our case, the patient was diagnosed with 21-OHD within 11 days, and subsequent treatment effectively prevented the development of severe phenotypes. Hormone replacement therapy is the primary treatment for CAH. However, as this is a long-term therapy, patients require regular monitoring of electrolyte levels, hormone levels, and growth/development parameters to prevent serious adverse effects. 28 Our case underscored the importance of timely diagnosis and intervention to rescue phenotypes and improve clinical outcomes. The goal of treatment is to correct adrenal insufficiency crisis and inhibit androgen synthesis, ensuring that growing individuals have as normal linear growth and puberty as possible. For patients who have developed, normal reproductive function needs to be maintained to the greatest extent possible. 29

## Conclusion

In conclusion, we presented a case with detailed clinical manifestations, follow-up through 12 months, and genetic diagnosis of classic CAH (SW form) attributable to 21-OHD and revealed the importance of early genetic diagnosis combined with timely therapeutic intervention and long-term monitoring. Through these approaches, the patient's phenotypes were effectively controlled and rescued. We further emphasized the incorporation of comprehensive genetic diagnostics (including WES, MLPA, Sanger sequencing, and LRS) in prenatal or postnatal diagnosis and provided classic educational value for CAH in infancy care.
