# Supplementary material for: Longitudinal 12-Month Follow-Up of a Male Infant with CYP21A2 Compound Heterozygous Genotype in China: A Case Report
Source: AJP Rep. 2025 Jul 18;15(3):e116–23. doi: 10.1055/a-2647-4369 (PMC12274093; doi:10.1055/a-2647-4369)
Supplement: Supplementary file 1 — Supplementary Material [file 10-1055-a-2647-4369_26603840.pdf]

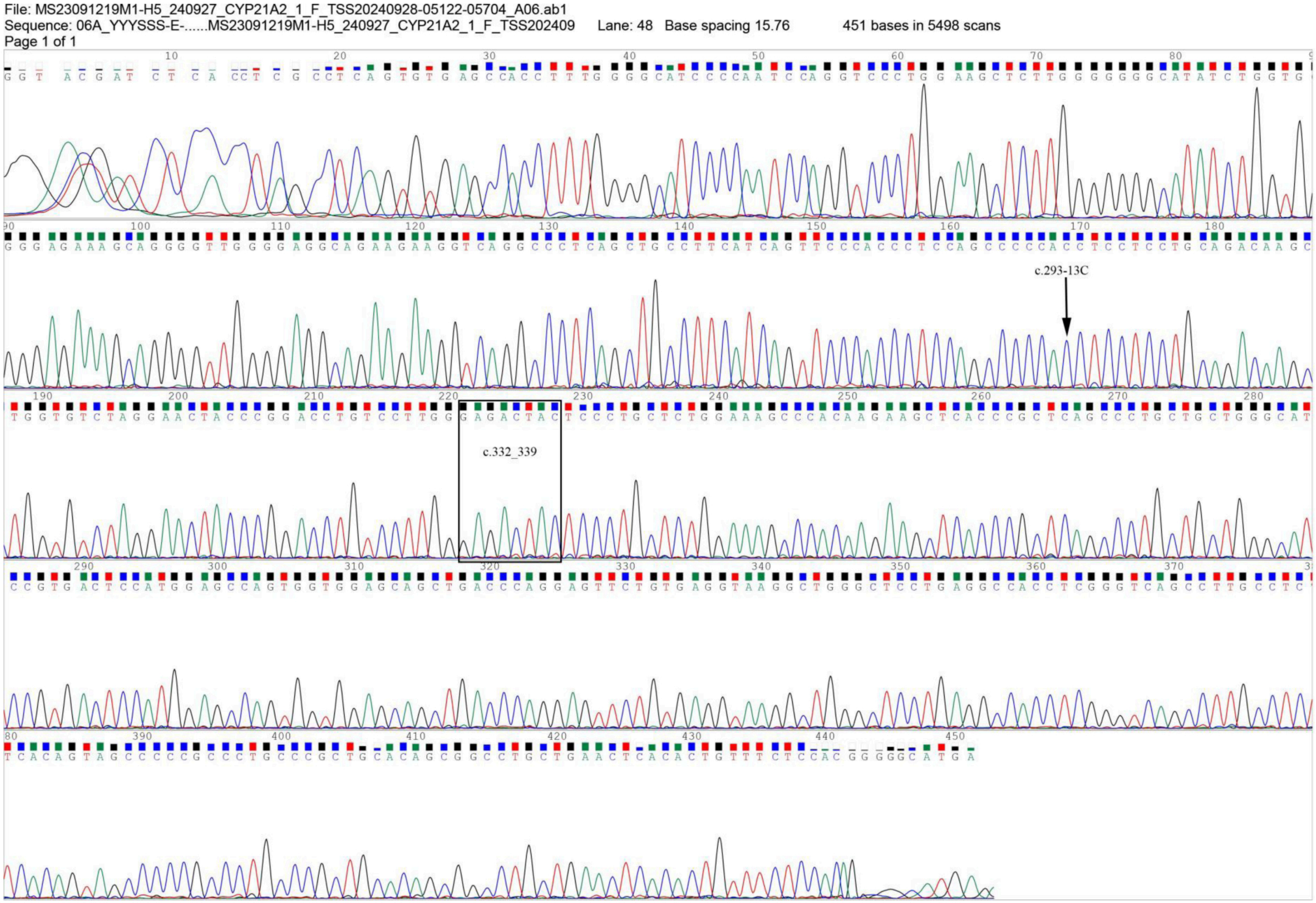
File: MS23091219M1-H5_240927_CYP21A2_1_F_TSS20240928-05122-05704_A06.ab1
Sequence: 06A_YYYSSS-E-......MS23091219M1-H5_240927_CYP21A2_1_F_TSS202409 Lane: 48 Base spacing 15.76 451 bases in 5498 scans
Page 1 of 1
c.293-13C
c.332_339

Mother

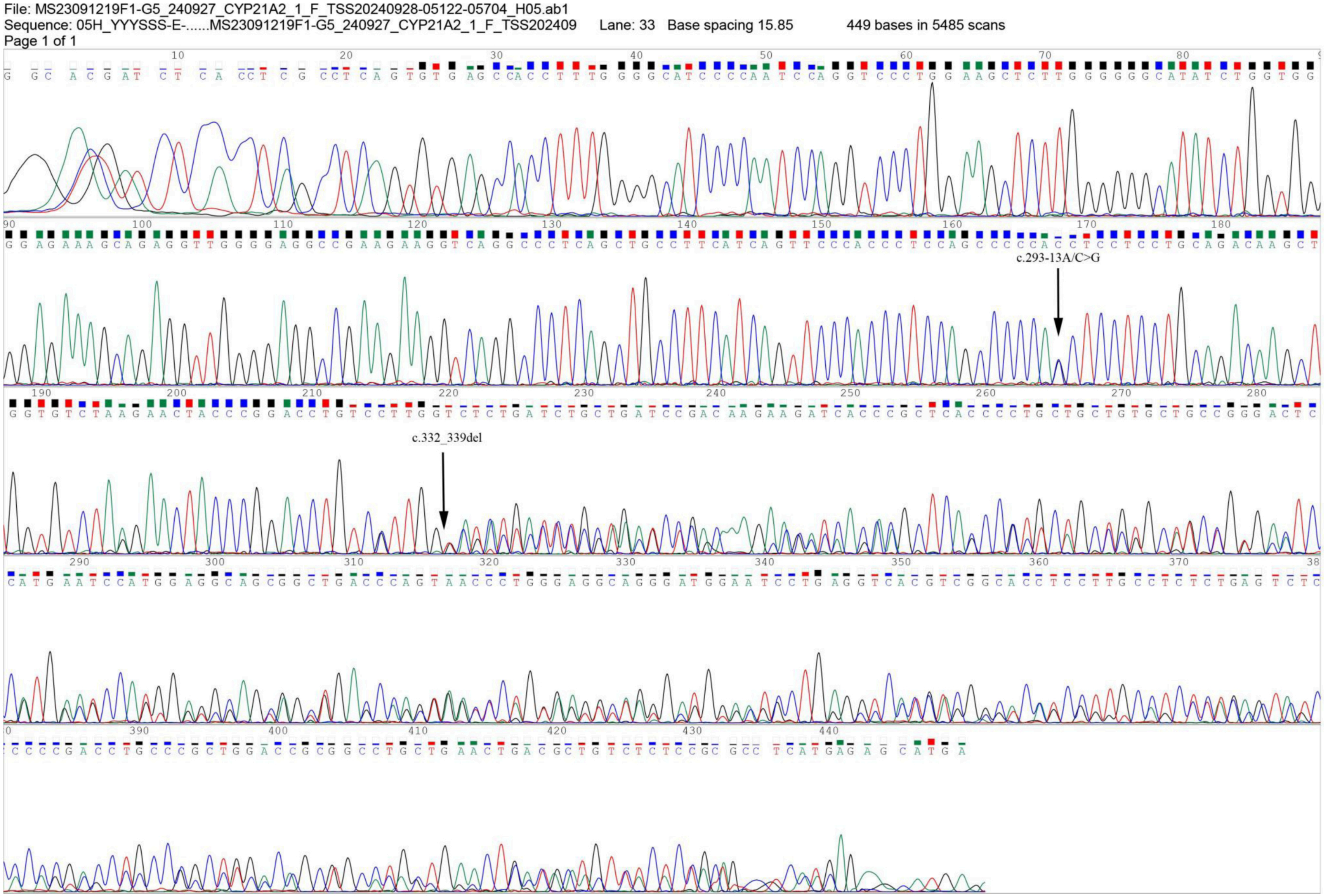
File: MS23091219F1-G5_240927_CYP21A2_1_F_TSS20240928-05122-05704_H05.ab1
Sequence: 05H_YYYSSS-E-......MS23091219F1-G5_240927_CYP21A2_1_F_TSS202409 Lane: 33 Base spacing 15.85 449 bases in 5485 scans
Page 1 of 1
c.293-13A/C>G
c.332_339del

Father

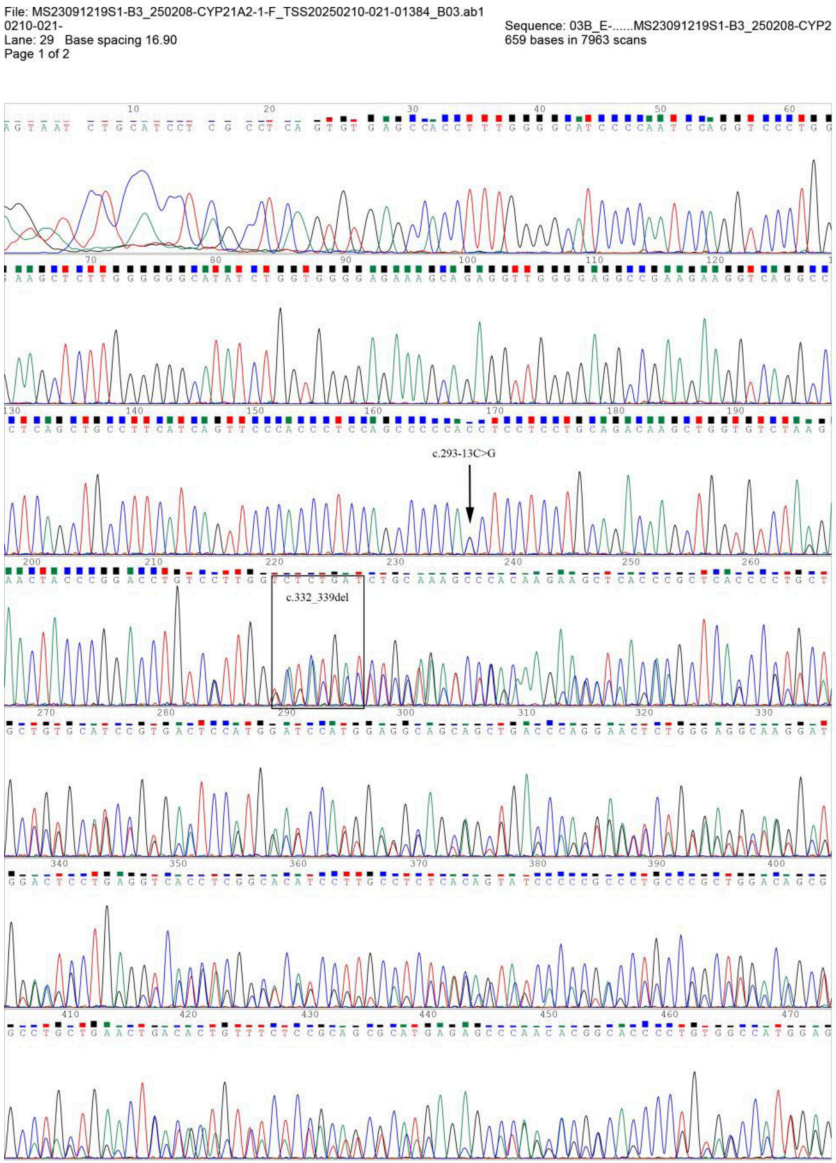

Grandparent I1

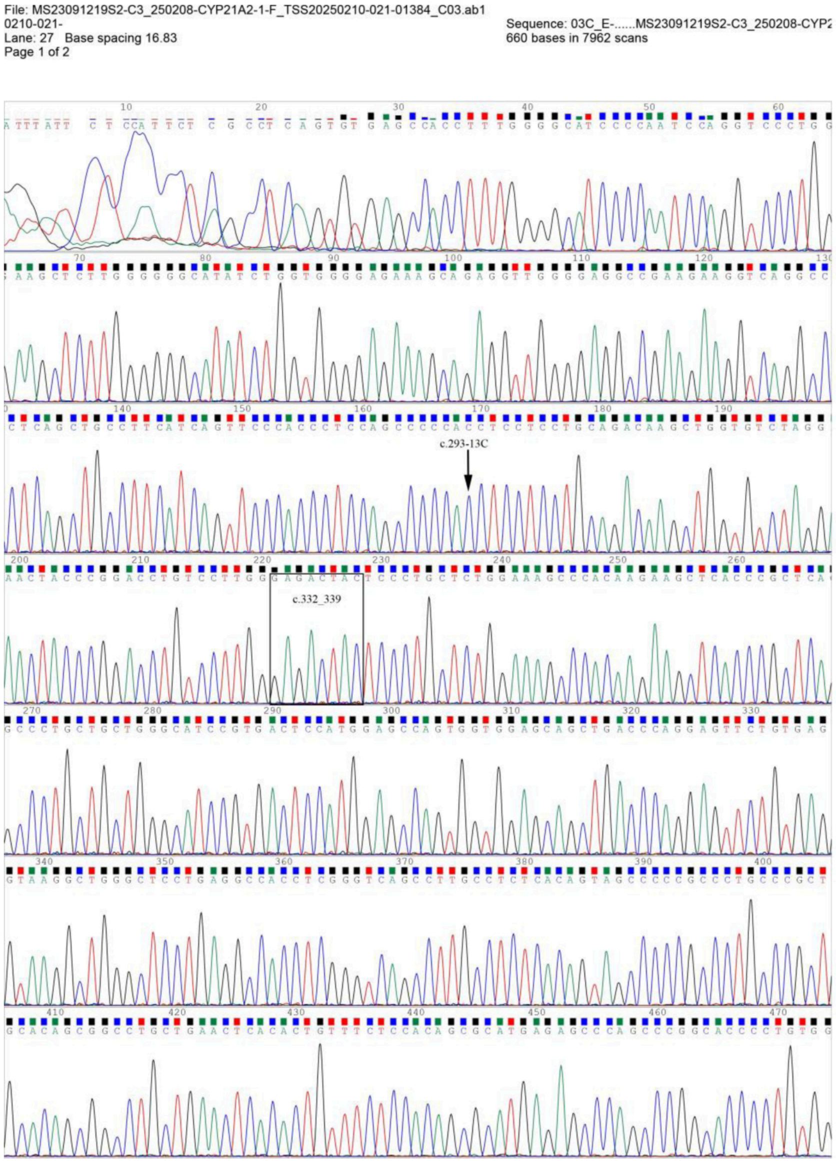

Grandparent I2

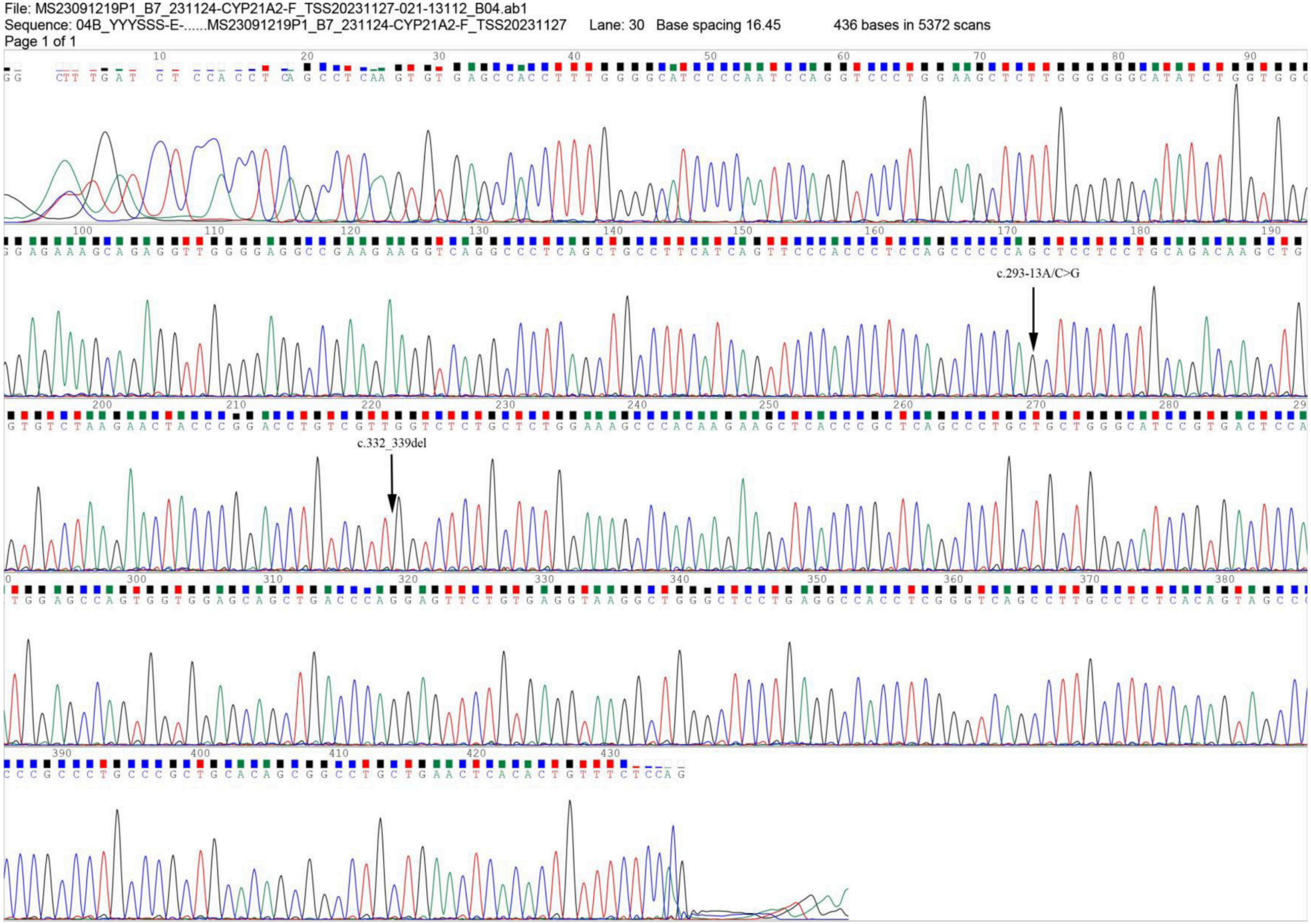
File: MS23091219P1_B7_231124-CYP21A2-F_TSS20231127-021-13112_B04.ab1
Sequence: 04B_YYYSSS-E-......MS23091219P1_B7_231124-CYP21A2-F_TSS20231127 Lane: 30 Base spacing 16.45 436 bases in 5372 scans
Page 1 of 1
c.293-13A/C>G
c.332_339del

Proband
